# Using functional traits to predict species growth trajectories, and cross‐validation to evaluate these models for ecological prediction

**DOI:** 10.1002/ece3.4693

**Published:** 2019-02-06

**Authors:** Freya M. Thomas, Jian D. L. Yen, Peter A. Vesk

**Affiliations:** ^1^ School of BioSciences, ARC Centre of Excellence for Environmental Decisions The University of Melbourne Melbourne Victoria Australia; ^2^ Present address: Interdisciplinary Conservation Science Research Group Centre for Urban Research, RMIT Melbourne Victoria Australia

**Keywords:** cross‐validation, ecological predictions, functional traits, growth models, non‐linear models

## Abstract

Modeling plant growth using functional traits is important for understanding the mechanisms that underpin growth and for predicting new situations. We use three data sets on plant height over time and two validation methods—in‐sample model fit and leave‐one‐*species*‐out cross‐validation—to evaluate non‐linear growth model predictive performance based on functional traits. In‐sample measures of model fit differed substantially from out‐of‐sample model predictive performance; the best *fitting* models were rarely the best *predictive* models. Careful selection of predictor variables reduced the bias in parameter estimates, and there was no single best model across our three data sets. Testing and comparing multiple model forms is important. We developed an R package with a formula interface for straightforward fitting and validation of hierarchical, non‐linear growth models. Our intent is to encourage thorough testing of multiple growth model forms and an increased emphasis on assessing model fit relative to a model's purpose.

## 1 INTRODUCTION

Growth in size through time is a fundamental biological process central to ecology. Growth rates define a relationship between size and age, which affect survival and fecundity of organisms (Arendt, 1997). Growth is complex, inherently variable, and is rarely maximized, which implies the existence of trade‐offs between growth and other important life history traits, such as fecundity (Arendt, 1997). Understanding plant trade‐offs is important for understanding variation in ecological strategies among species, which in turn allows one to make generalizations across species. The ability to quantitatively generalize across plant species (Harper, 1967) has progressed with the development of functional trait‐based schemes (Keddy, 1992; Falster et al., 2016; Levine, 2015; Westoby, 1998; Westoby, 1999). Leaf and wood density functional traits have been compiled for thousands of species across hundreds of sites worldwide (Chave et al., 2009; Wright et al., 2005) and compilations of other trait databases has culminated in the global plant traits database TRY which includes 93 trait databases and millions of trait entries across thousands of plant species worldwide (Kattge et al., 2011).

An exciting prospect of large amounts of readily available functional trait data is to apply trait‐based generalizations and predictions to applied management problems. In these problems, time, money, and data are often limited; yet, decisions must be made across broad suites of species. Size‐structured dynamics are thought to be particularly important in understanding population and vegetation dynamics over both short and long time scales (Arendt, 1997; Falster & Westoby, 2005) and have useful applications to environmental management decisions (Muir, Vesk, & Hepworth, 2014; Munro, Fischer, Wood, & Lindenmayer, 2009), particularly those underpinned by conceptual ideas about species’ life history strategies and their responses to disturbance (Driscoll et al., 2010; Noble & Slatyer, 1980). For example, decisions about when to burn ecosystems in fire‐prone regions are often based on limited information (Bradstock & Kenny, 2003; Cheal 2010; Driscoll et al., 2010), in particular limited species‐specific data (Keith, 2012). Plant height has been used as an indicator of habitat after fire (Haslem et al., 2012, 2011 ) and to inform tolerable fire intervals (Muir et al., 2014).

Trait‐based models of growth are a promising tool that can use large amounts of readily accessible functional trait data to predict life‐history aspects of vegetation (Rüger et al., 2011; Falster, Duursma, & FitzJohn, 2018), which is useful for environmental management. However, before trait‐based models can be applied widely, thorough interrogation and hard tests of the predictive capacity of this approach is necessary (Visser et al. 2016). Models of individual growth range from simple calculations of relative growth rates (Poorter, 1989) to complex mechanistic models (Falster, FitzJohn, Brännström, Dieckmann, & Westoby, 2016; Prusinkiewicz, 2004). Although some very large data sets support complex models (Rüger et al., 2011), many empirical studies generate small data sets to test novel ideas (Falster & Westoby, 2005) or to guide management decisions (Muir et al., 2014; Munro et al., 2009). These small data sets are often unsuited to highly complex models (Buckland, Burnham, & Augustin, 1997). Parametric growth models are well suited to model fitting when few data are available (Paine et al., 2012). The use of semi‐rigid model forms acknowledges prior information about expected growth trajectories, and assumes commonality among individuals of a species and commonalities in the processes influencing growth of individuals. An extensive literature describes different non‐linear model forms and various parameterizations (for reviews, see Paine et al., 2012; Pommerening & Muszta, 2016; Zeide, 1993). Much of this literature emphasizes technical model details (Hunt, 1982; Tsoularis & Wallace, 2002) alongside explanations of the biological relevance of different model forms (Tjørve 2003, 2009 ; Tjørve & Tjørve, 2010a; Tjørve & Tjørve, 2010b). Some studies provide general guidance on model selection and the calculation of common outputs from growth models, such as relative and absolute growth rates (Huang, Titus, & Wiens, 1992; Paine et al., 2012; Vanclay & Skovsgaard, 1997; Yang & Huang, 2011).

Many different statistical growth model forms exist (Tjørve, 2009). Some authors suggest little difference between model forms, with most tested non‐linear models performing well for growth analysis (Arabatzis & Burkhart, 1992; Zwietering, Jongenburger, Rombouts, & Van't Riet, 1990). Comparisons and evaluation of nonlinear growth models often use information criteria (e.g., AIC, DIC, WAIC), which can be used to rank models based on relative information loss (Burnham, Anderson, & Huyvaert, 2011; Hooten & Hobbs, 2015; Stephens, Buskirk, & Rio, 2007; Symonds & Moussalli, 2011). Although information criteria can identify the top‐ranked model among a set of possible models, they do not provide absolute estimates of model fit and do not necessarily contain information on how a model will perform for a particular task (e.g., prediction; Mac Nally, Duncan, Thomson, & Yen, 2018).

Increasingly, it is recognized that performance should be measured relative to a model's intended application (Mac Nally et al., 2018; Symonds & Moussalli, 2011). One common application of growth models is prediction, which allows information on growth to be transferred to new locations or species (Rüger et al., 2011; Thomas & Vesk, 2017b). Predictive tests of models can identify over‐specified explanatory models, and predictions directly support the development of new hypotheses. One can construct predictive growth models by incorporating predictor variables into a hierarchical modeling framework (Camac, Williams, Wahren, Hoffmann, & Vesk, 2017; Pollock et al., 2012; Rüger et al., 2011; Thomas & Vesk, 2017b). Here, our interest is predicting entire species height‐growth curves from traits using data collected over chronosequences of time‐since‐disturbance (Falster & Westoby, 2005; Muir et al., 2014; Thomas & Vesk, 2017a, 2017b). Predicting growth for new species, as opposed to individuals of the same species, is an emerging field (Rüger et al., 2011; Uriarte, asky, Boukili, & Chazdon, 2016; Visser et al. 2016). To predict to new species, models must capture the most relevant traits and growth processes and, as such, pushing models to predict to out‐of‐sample data and across multiple species is likely to increase mechanistic understanding of trait‐growth relationships (Falster et al., 2018).

Similar to the selection of a particular model form, identifying the appropriate predictor variables to include in a given model is difficult and there is no general consensus on how to select appropriate variables (Allen, 1971; Hooten & Hobbs, 2015). Two options are to select a subset of predictor variables or to build a “global model” with all variables included (Burnham et al., 2011; Mac Nally, 2000;). One major benefit of global models is that they circumvent the need for variable selection (Burnham et al., 2011). However, including all variables can lead to lack of parameter precision and over‐parameterization, which can generate spurious predictive results (Mac Nally, 2000). A common recommendation in model building is to limit model complexity (Paine et al., 2012), which may suggest choosing a growth model with fewer parameters as well as limiting the overall number predictor variables included in a given growth model.

Several evaluation techniques exist for predictive models, including cross‐validation (Allen, 1971; Michaelsen, 1987; Stone, 1974) and out‐of‐sample prediction (Fleishman et al., 2018; Mac Nally, 2000). Cross‐validation is an in‐sample method that asks whether a model will generalize to other data from the same statistical population. Out‐of‐sample prediction asks whether a model will generalize to a new statistical population. Here, we demonstrate a form of stratified cross‐validation (Roberts et al., 2017), where we ask whether the fitted model will generalize to an unobserved species from the same statistical population. This is a relatively harsh test of predictive performance, but relates directly to our objective of predicting species information from functional traits.

The distinction between choosing a model based on fit to the underlying data and choosing a model based on predictive capacity is often ignored (Burnham et al., 2011; Hooten & Hobbs, 2015; Mac Nally et al., 2018). While several information criteria such as AIC are asymptotically equivalent to leave‐one‐out cross‐validation, such measures do not provide an absolute assessment of model fit and are not reliable for some ecological data sets where data are scarce (Mac Nally et al., 2018). Given the importance of predictions in many ecological applications, we believe that model evaluation is crucial. However, the “correct” evaluation statistics seem to change as new packages and programs become available, and out‐of‐sample prediction is rarely emphasized in the ecological literature (but see Hooten & Hobbs, 2015; Thomas & Vesk, 2017b; Zhang, 1997).

There are few practical examples of robust evaluation in the ecological non‐linear modeling literature. We hope to contribute to the practice of growth modeling by developing methods and code for the evaluation of predictive capacity of non‐linear growth models. We construct predictive height‐growth models based on traits, compare 11 different forms of growth model, compare models with selected variables or all variables, and compare cross‐validated and out‐of‐sample measures of model fit. This paper is accompanied by an R package, growmodr, to fit and validate non‐linear growth models (available at https://github.com/jdyen/growmodr). An example of fitting and validating a growth curve model is in Supporting Information Appendix S1.

## 2 METHODS

### 2.1 Height growth and functional trait data sets

We demonstrate the usefulness of interrogating multiple growth model forms and using out‐of‐sample cross‐validation with a case study on three independently collected data sets from south‐eastern Australia. Each data set contains data on heights of individuals from non‐resprouting plant species over a chronosequence of sites with different time‐since‐fire from three ecosystems in south‐eastern Australia. These datasets contain growth data at the species level, that is, size‐at‐age data. The mallee dataset contains 15 species (923 individual heights) from a low open mallee woodland in Murray Sunset National Park Victoria; the Myall dataset contains 16 species (506 individual heights) from a coastal open woodland in Myall Lakes National Park New South Wales; the Foothills dataset contains nine species (360 individual heights) from a damp forest in Foothills Forest around Melbourne Victoria. These datasets also report functional traits collected from individuals and averaged for each species at each site following established protocols (Cornelissen et al., 2003). See Supporting Information Appendix S2: Table S5 for species lists. Each study used a chronosequence approach to collect height data over time‐since‐fire sites and within each study only woody species occupying similar edaphic conditions were sampled; details of sampling methodologies are in (Falster & Westoby, 2005; Muir et al., 2014; Thomas & Vesk, 2017b). While we focus on heights of woody plants for our case studies, the methods could relate to other data such as growth in length or mass of fishes (Morrongiello & Thresher, 2015).

### 2.2 Growth models

We fitted 11 non‐linear growth models to height data from each species (Table 1). We used a hierarchical model structure to model all species simultaneously and relate the parameters of the growth models to traits. We have previously used this modeling approach to study interspecific height races between plants in a semiarid fire‐prone ecosystem (Thomas & Vesk, 2017b) and to assess the transferability of our trait‐based models between ecosystem types (Thomas & Vesk, 2017a). The incorporation of traits as predictor variables into non‐linear growth models for multiple species allows predictions of growth among species (Pollock et al., 2012). Traits could be replaced by any species‐ or individual‐level predictor variable of interest (e.g., environmental conditions, phylogeny). Our use of functional traits supports predictive models of plant growth through time for multiple species, and also gives mechanistic insight into the contributions of functional traits to inter‐ and intra‐specific variation in growth.

**Table 1:** eleven non‐linear growth model forms. All have 2–3 parameters models and can be classified as concave or sigmoidal and as bounded or unbounded

Model name	Abbrev.	Parameters	Model type	Model equation
Exponential	EXP2	2	Concave; unbounded	$\alpha +\beta \log\left(x\right)$
Kobayashi logarithmic	KOBLOG2	2	Concave; unbounded	$a\log\left(1+\frac{x}{\beta }\right)$
Monod	MONOD2	2	Concave; bounded	$\alpha \left(\frac{x}{\beta +x}\right)$
Negative exponential	NEGEXP2	2	Concave; bounded	α(1−exp−βx)
Power	PWR2	2	Concave; unbounded	$\alpha x^{\beta }$
Extended Power	PWR3	3	Concave; unbounded	$\alpha x^{\beta -(\omega /x)}$
Archibald	ARCH3	3	Sigmoidal; bounded	$\frac{\alpha }{\left(\beta +\omega^{x}\right)}$
Hillslope	HS3	3	Sigmoidal; bounded	$\frac{\alpha }{\left(1+\exp(-\beta (x-\omega ))\right)}$
Hillslope log	HSlog3	3	Sigmoidal; bounded	$\frac{\alpha }{\left(1+\exp(-\beta (\log(x)-\omega )))\right)}$
Logistic	LOG3	3	Sigmoidal; bounded	$\frac{\alpha }{\left(1+\exp(-\beta x+⊖\omega )\right)}$
Cumulative Weibull	WEIB3	3	Sigmoidal; bounded	$\alpha (1-\exp\left(-\beta x^{\omega }\right))$

Our models had a hierarchical structure that accounted for variation among species and among individuals. Hierarchical models allow explained and unexplained variation to be partitioned within and among multiple levels of a dataset (in this case, species and individuals). Parameters can differ among levels under an assumption that they are drawn from a common distribution (Condit et al. 2006; Gelman and Hill 2007; Rüger et al., 2011). The advantage of the hierarchical approach is that information can be shared among species and individuals so that parameters for rare or data‐scarce species can be informed by parameters of other more data‐rich species. This is beneficial when field data are scarce for many species but when we (as ecologists) wish to include all species in our analysis, including those with few observations. However, caution is necessary because hierarchical models can potentially make rare species appear similar to more common species.

We used a lognormal observation model to model the heights of individual plants (see Equation ((1)), below). The lognormal distribution reflects natural constraints on height data, which take positive values with few extreme height values (Limpert, Stahel, & Abbt, 2001). We modeled the mean of the lognormal distribution with one of eleven non‐linear growth models, so that mean heights of individuals were mathematical functions of age, with one or more parameters for each function (Table 1). For a given non‐linear growth model, we related each parameter to a set of species‐level traits that were posited to affect growth dynamics (Equation ((2)). We used linear models to relate growth‐model parameters to traits, and included species‐specific trait effects and intercepts (Equation ((3)). We evaluated models at the species level; this process is described in more detail below (see Model evaluation: “naïve” model fit vs. “*n*‐species‐fold” cross‐*validation*, below).

We used hypothesized and observed trait‐growth relationships from past studies to decide which functional traits to include in our models (Falster & Westoby, 2005; Moles & Westoby, 2006; Reich, 2014; Sterck, Poorter, & Schieving, 2006; Thomas & Vesk, 2017a, 2017b). Based on these studies, we hypothesized that seed mass would strongly influence initial growth, stem density would influence achievable height, and leaf traits would influence the whole growth process (Thomas & Vesk, 2017a). We include the parameterization of each model in Supporting Information Appendix S2: Table S4.

**Figure 1:**
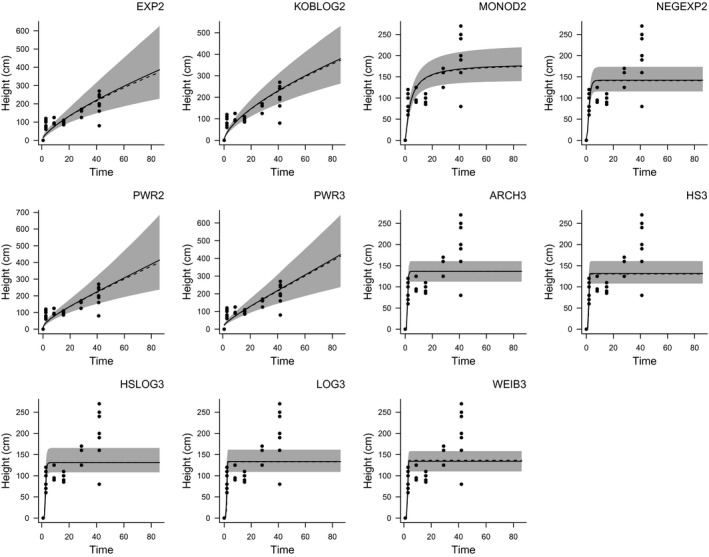
The shape of each of our eleven tested growth models fitted to one species (*Acacia brachybotrya* (Mimosaceae)) from the mallee data set. Axes extend beyond the observed data because the predictions extend to the maximum time‐since‐fire for any species in the mallee data set (86 years). Black lines are mean model predictions, dashed lines show the median

We describe each nonlinear growth model in Table 1 and show an example of the model form for one species in Figure 1. The model equations are:

(1) $$H_{i,j}∼\text{lognormal}\left(\mu_{i,j},\;\sigma_{i}\right);\;$$

(2) $$\mu_{i,j}=f\left(x_{i,j}\beta_{1,j},\beta_{2,j},\beta_{3,j}\right);$$

(3) $$\beta_{k,j}=\sum_{G}\gamma_{k,g,j}t_{g,j},$$

where *H_i,j_* is the observed *i*th height data point for species *j*,* μ_i,j_* is the mean height (on a log scale) of species *j* at age *x_i,j_*,* β_k_* are the parameters of a given growth model *f*(˙·), which takes one of the model forms listed in Table 1, and *γ_k,_*_g_ *_,j_* is the association between the growth‐model parameter *β_k_* and trait *g*, which takes value *t_g,j_* for species *j*. We allowed the set G of all traits to differ among growth‐model parameters (Appendix S2: Table S4).

### 2.3 Model fitting

We used Bayesian inference to estimate model parameters. Specifically, we used Hamiltonian Monte Carlo (HMC) implemented in the open‐source software package Stan version 2.12.0 (Stan Development Team, 2016a; Stan Development Team, 2016b). We handled all data and Stan model outputs in the statistical software environment R version 3.4.0 (R Development Core Team 2015) with the package rstan (Stan Development Team, 2016a; Stan Development Team, 2016b). Standard deviations for the observation error and random effects were modeled as positive half‐normal distributions, with prior mean 0 and prior standard deviation 2 (Gelman, 2006). We initialized our model using random values drawn from the prior distributions. We provide example code and data to conduct evaluation under naïve fitting and internal cross‐validation in the Appendix S1 for a simple dataset. We include all methods in the R package growmodr, available at https://github.com/jdyen/growmodr. We include example code in Appendix S1.

### 2.4 *Model evaluation: “naïve” model fit* versus* “n‐species‐fold” cross‐validation*

We compared each of our growth models for all three data sets with evaluation statistics calculated in two ways in order to compare between “naïve” and “*n*‐species‐fold” cross‐validation. First, we used all data within each data set to train a given model and compared fitted and observed height values using three evaluation metrics (see *Model evaluation statistics*, below). We refer to this method of model evaluation as “naïve” model fit. Second, we used *n*‐species‐fold internal cross‐validation: for each data set the height data for one species was removed from the training data, and the fitted model was used to predict the height of the removed species. This process was repeated for every species. We used species‐based cross‐validation because our interest is predicting entire species growth curves from traits (see Thomas & Vesk, 2017a, 2017b); this is a form of stratified cross‐validation (Roberts et al., 2017).

### 2.5 Model evaluation statistics

**Figure 2:**
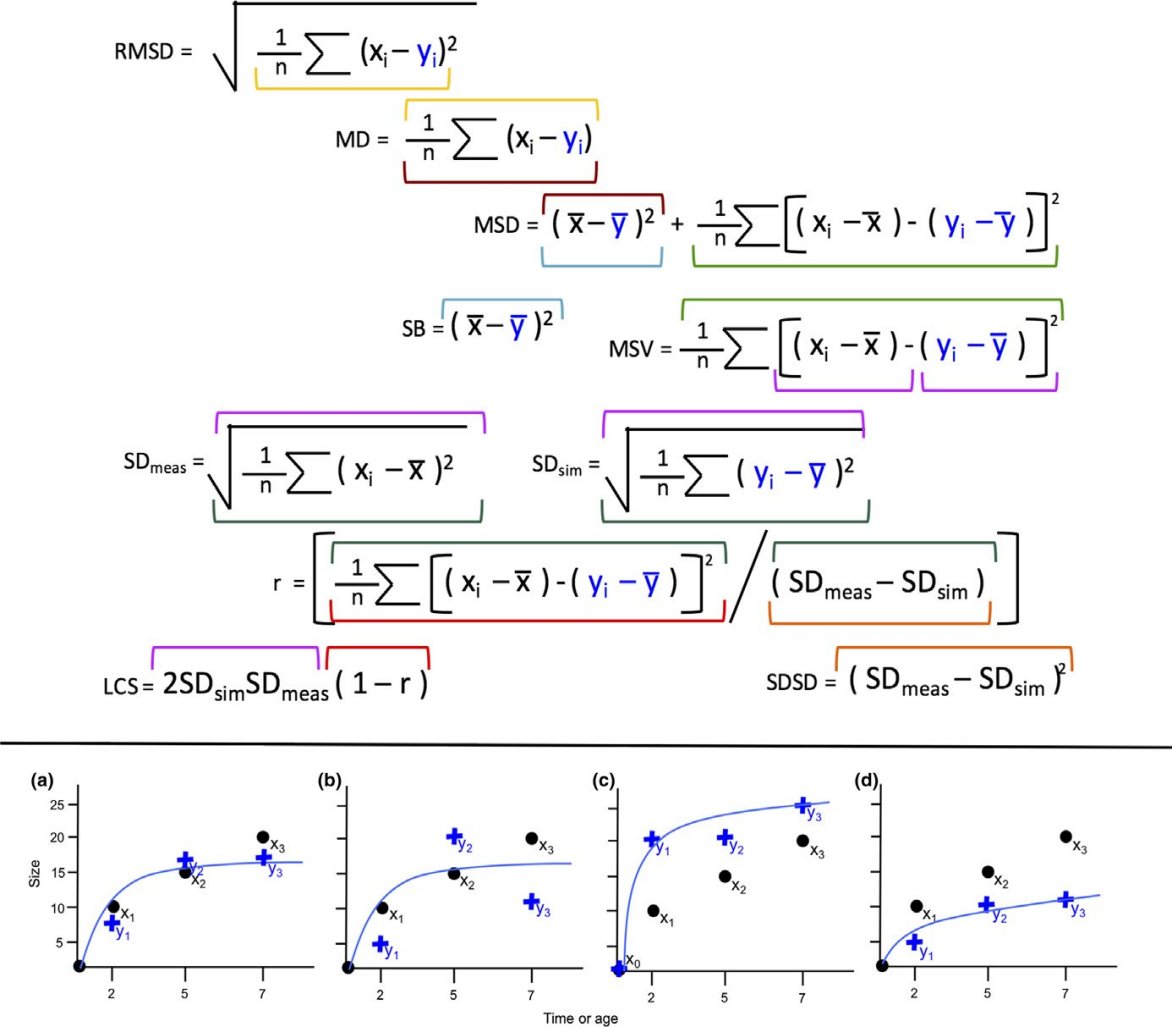
Related metrics that use a comparison between observed (*x_i_*) and predicted (*y_i_*) data (Kobayashi & Salam, 2000). Colors show the related parts of each equation. Root mean squared deviance (RMSD); mean deviance (MD); mean squared deviance (MSD); simulation bias (SB); mean squared variation (MSV); standard deviation of the measurement (SDmeas) and standard deviation of the simulation (SDsim); the correlation coefficient (*r*); the lack of positive correlation weighted by the standard deviations (LCS); and the difference in magnitude of fluctuation between simulation and measurement (SDSD). The bottom panels (a–d) provide hypothetical examples of different observed (black) and predicted values (blue), showing (a) close relationship that would have a high *r* ^2^, low RMSD, and low MD, (b) a relationship lacking precision that would have a good *r* ^2^, high RMSD but low MD, (c) a relationship displaying over‐prediction that would have a good *r* ^2^, moderate RMSD, and high positive MD; and (d) a relationship showing under‐prediction that would have a good *r* ^2^, moderate RMSD, and high negative MD

We used three model evaluation statistics to calculate model fit: Pearson's correlation coefficient (*r* ^2^), root mean squared deviance (RMSD), and mean deviance (MD). All three evaluation statistics are based on the difference between the observed data (*x_i_*) and the predicted data from the model (*y_i_*; Figure 2). Many related metrics could be used (Figure 2); we use these three because they represent the amount of variation explained by a given model, how accurate a given model is, and how biased a given model is, respectively. Kobayashi and Salam (2000) and Gauche et  al. (2003) contain further discussion on these metrics.

Pearson's correlation coefficient (*r* ^2^), also known as the coefficient of determination, is a measure of the proportion of variation explained by a given model. An *r* ^2^ of one indicates that model predictions exactly match our observed data. This approach assumes that data are continuous and there is a linear association between predicted and fitted values. Root mean squared deviance (RMSD) is a measure of accuracy, defined as the square root of the mean of the squared differences between observed data and model predictions. Lower values of RMSD indicate more accurate models (Kobayashi & Salam, 2000; Wright et  al., 2005). Mean deviance (MD) is a measure of model bias, defined as the average difference between the mean model prediction and the observations (Kobayashi & Salam, 2000; Figure 2). Positive values of MD indicate over‐prediction and negative values indicate under‐prediction (Figures 2c–d).

There are no accepted conventions for identifying good versus poor model fit based on these metrics, that is, the value of a metric (e.g., RMSD) that describes a good model fit compared to a bad model fit. Decisions on what constitutes a good or bad model fit should be based on the study objectives and biological effect sizes, and will depend on the consequences of a poor model. For example, a growth model with a positive bias of 2 m would be a bad model for a shrub species with an average maximum height of 1 m. By contrast, a 2 m bias would be negligible if our aim was to predict the height of the tallest flowering plant in the world (*Eucalyptus regnans*), which can reach maximum heights of 95 m.

We used thresholds based on expected height values of plants across three‐different metrics relating to explained variation, bias and accuracy to delimit models into four categories of performance (“good,” “adequate,” “poor,” and “bad”). We used subjective thresholds instead of raw evaluation metrics to provide a comprehensible summary of large amounts of information and additionally to highlight that model evaluation is subjective based on the objective of the modeling exercise (Mac Nally et al., 2018). We do not want a well‐fitted model representing a simplification of our ecological system that produces biologically meaningless results (Mac Nally, 2000; Roberts et al., 2017; Symonds & Moussalli, 2011). In general, we would expect cross‐validated performance to be lower than naïve performance because the model is trained on fewer data and predictions are compared to observations that the model has not been exposed to. In order to compare performance between naïve model fit and cross‐validation, we used different thresholds of model fit for naïve and cross‐validated models. RMSD and MD have units of cm (the height data are in cm), so a MD > 500 is equivalent to the model predicting heights biased by 5 m. We would expect that a good model fit, based on cross‐validation, would have the highest *r* ^2^ and an overall variation around the mean modeled height of <1 m (RMSD) and the bias (MD) in predicted heights should be <0.5 m in either direction. We conducted a sensitivity analysis by doubling and halving our chosen thresholds to see whether this altered our inferences; the ranks of each model remained the same when thresholds were doubled or halved. Table 2 outlines each threshold for each metric between naïve and cross‐validated performances, we also provide a written example for clarity.

**Table 2:** Subjective thresholds for metric performance based on modeled height data. We expect cross‐validated performance to be lower than naïve performance because the model is trained on fewer data and predictions are compared to observations that the model has not been exposed to. In order to compare performance between naïve model fit and cross‐validation, we used different thresholds of model fit for naïve and cross‐validated models

Metrics	“Good”	“Adequate”	“Poor”	“Bad”	Written definition of “Good”
$r_{na\ddot{i}ve}^{2}$	>0.75	0.40–0.75	0.10–0.40	<0.10	For naïve statistics, a good R2 means more than 75% of the variation in height is explained by the fitted model.
RMSD_naïve_	<50	50–100	100–200	>200	For naïve statistics, a good RMSD means the average variation around the mean fitted height at any time is <50 cm
MD_naïve_	<10	10–30	30–100	>100	For naïve statistics, a good MD means the average bias in modeled height measurements is <10 cm
$r_{\text{cv}}^{2}$	>0.40	0.20–0.40	0.10–0.20	<0.10	For cv leave‐one‐species‐out statistics, a good R2 means more than 40% of the variation in height is explained by the fitted model
RMSD_cv_	<100	100–200	200–500	>500	For cv leave‐one‐species‐out statistics, a good RMSD means the average variation around the mean fitted height at any time is <100 cm
MD_cv_	<50	50–100	100–500	>500	For cv leave‐one‐species‐out statistics, a good MD means the average bias in modeled height measurements is <50 cm

Our results have many components; eleven models, three data sets, and three evaluation metrics, each of which is compared with naïve and cross‐validated evaluation statistics. This process was repeated with each data set either including all plant traits as predictor variables or with a selected subset of traits as predictor variables. To visualize broad trends in our results, we summarized a model's overall performance based on consensus among the evaluation metrics: if two or three of the evaluation metrics were in one category (i.e., good, adequate, poor, or bad), we concluded that the model's overall performance was in that category. If all three metrics differed, we chose the middle category, and if all three metrics were the same the model performance was that category. For example, if model performance was “poor” for two metrics and “bad” for the other, we defined that model's performance as “poor.” In order to visualize this information, we constructed color‐coded tables. The raw numbers and the magnitude of difference between metrics are important for robust ecological interpretations of fitted models, and we present the raw values in the (Appendix S2: Tables S1–S3).

## 3 RESULTS

### 3.1 Can traits predict tree growth curves?

**Figure 3:**
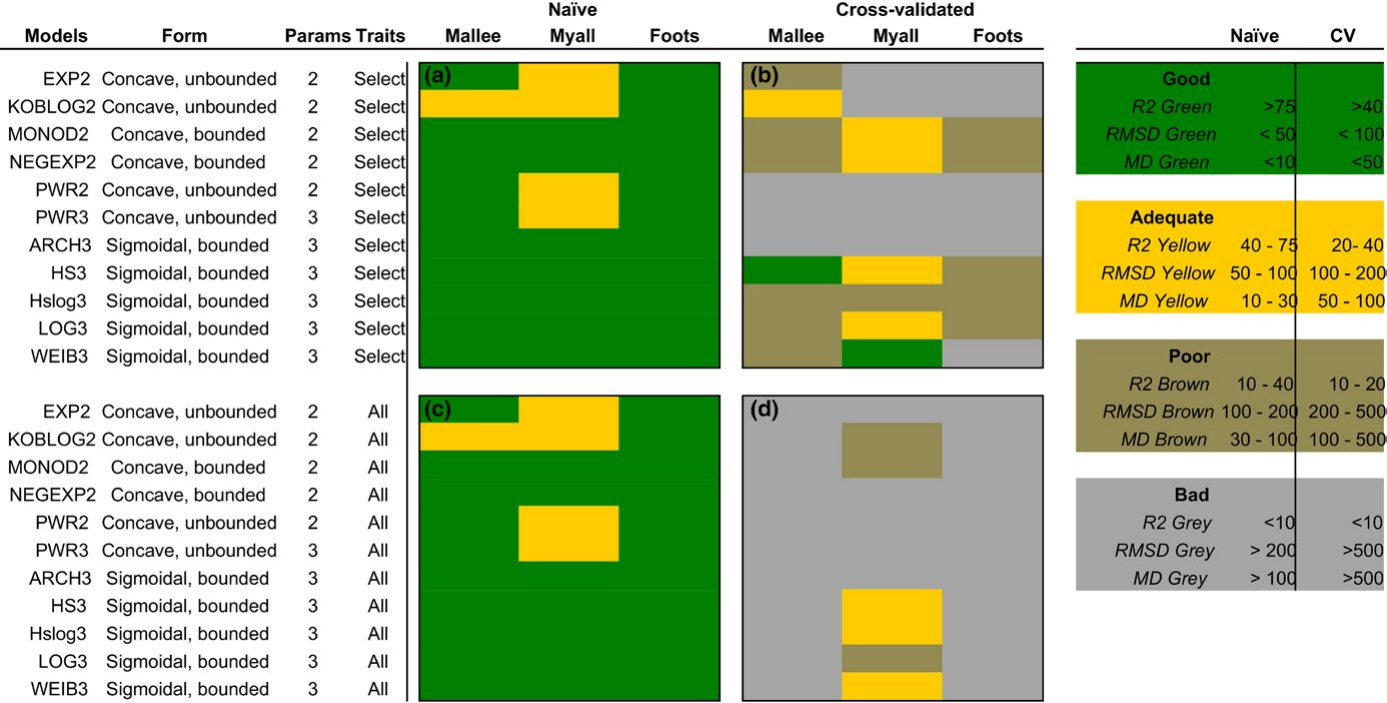
Overall model performance was established as a consensus across all three metrics used. Here, we show this consensus model performance among data sets, between global (panels c and d) and specific‐trait (panels a and b) models, and between naïve (panels a and c) and cross‐validated (panels b and d) evaluation metrics. The legend displays color‐coded thresholds that distinguish “good” (green), “adequate” (yellow), “poor” (brown), and “bad” (gray) metric performance. Note different thresholds between naïve and cross‐validated performance metrics (explained in text)

There was on average little difference in naïve *r* ^2^ values ($r_{na\ddot{i}ve}^{2}$) between “global models” (models based on all traits) and our “specific‐trait models” (models based on an ecologically relevant subset of traits; Figure 3). Comparing cross‐validated *r* ^2^ values ($r_{\text{cv}}^{2}$) between global and specific‐trait models, global models performed consistently worse than the specific‐trait models. Not one of the global models performed as well as the best performing specific‐trait model under internal cross‐validation (hillslope with $r_{\text{cv}}^{2}$ of 0.434; Figure 3). Hence, for cross‐validated model performance, including all traits leads to mostly “bad” model performance, while using only a subset of traits leads to some “good” and “adequate” model performances (Figure 3). We focus on the specific‐trait models in the remainder of this section.

All specific‐trait models fitted the three data sets well, with $r_{na\ddot{i}ve}^{2}$ values between 0.708 and 0.884 (Figure 3, Appendix S2: Tables S1–S3). However, naïve and cross‐validated statistics differed markedly; naïve statistics had higher $r_{na\ddot{i}ve}^{2}$, lower root mean squared deviance and lower mean deviance across all models (Figure 3). For example, for the specific‐trait model in the mallee dataset, naïve *r* ^2^ values were on average 64 times larger than cross‐validated *r* ^2^ values (ranging from a twofold to a 376‐fold difference), naïve precision (RMSD) values were on average seven times smaller than cross‐validated precision values (ranging from a twofold to a 29‐fold difference), and naïve bias was on average 55 times smaller than cross‐validated bias (ranging from equal to a 258‐fold difference) (excluding models for which metrics were incomparable [i.e., infinite values]; Appendix S2: Table S1). One of the best fitting models based on $r_{na\ddot{i}ve}^{2}$ was the three‐parameter logistic ($r_{na\ddot{i}ve}^{2}$ = 0.879), which had one of the lowest performances under cross‐validation ($r_{na\ddot{i}ve}^{2}$ = 0.022; Appendix S2: Table S2).

**Figure 4:**
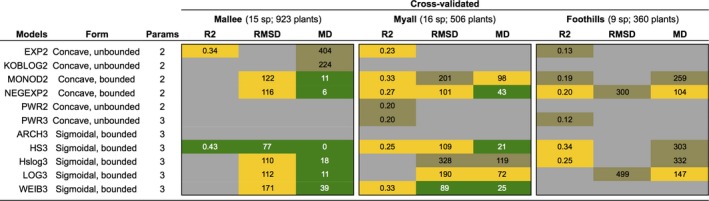
Performance metrics for each data set calculated from cross‐validated, specific‐trait models (detailed and unpacked version of top left panel of Figure 3). Metric thresholds are as described in the methods and shown in legend of Figure 3. Values for gray shaded boxes are not reported for ease of viewing; these values were typically large or infinite for RMSD and MD and zero for *r* ^2^. Full results are in Appendix S2: Tables S1–S3

Performance metrics for the cross‐validated, specific‐trait models differed within and among data sets (Figure 4). No single model was best across all data sets (Figure 4). In the mallee data set, the hillslope was the only model with $r_{\text{cv}}^{2}$ > 0.4, the two‐parameter exponential had an adequate $r_{\text{cv}}^{2}$, and the remaining models performed poorly with $r_{\text{cv}}^{2}$ ranging from 0–0.08. The two other evaluation metrics (RMSD, MD) ranked models similar to this. In the Myall data set, the best‐performing model was the three‐parameter Weibull, and in the Foothills data set, the hillslope and the two‐parameter negative exponential performed well, as did the three‐parameter logistic (Figure 4).

Based on the cross‐validated, specific‐trait model performance metrics (top right panel, Figure 4), and rejecting models with any “bad” performance across all three data sets, the best‐performing models were the hillslope, log hillslope, three‐parameter logistic, two‐parameter monod, and two‐parameter negative exponential. These model forms all include an asymptote for maximum height. Removing models without “adequate” performance in at least two datasets, the hillslope model was the best‐performing model overall (Figure 4).

### 3.2 How do different model evaluation statistics compare?

**Figure 5:**
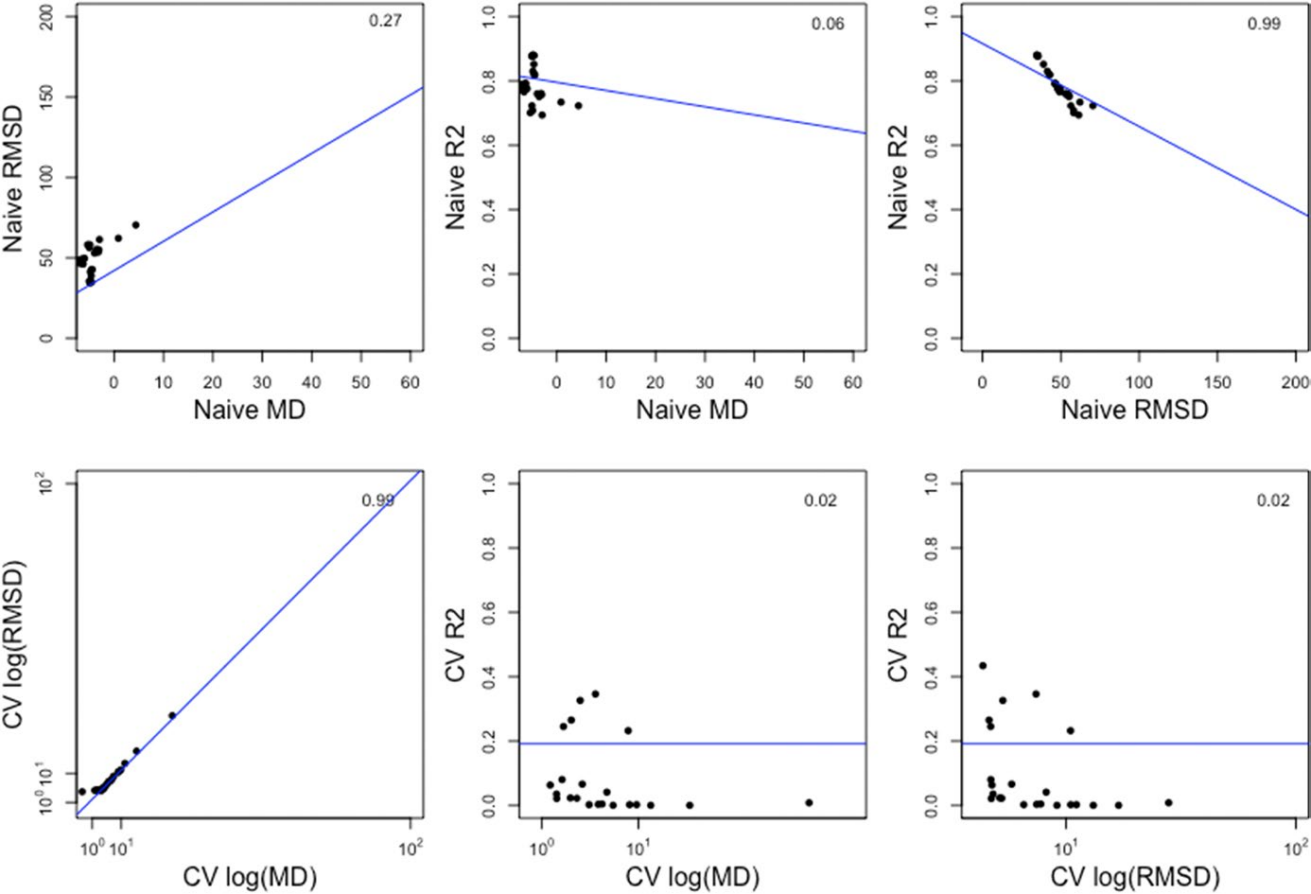
Relationships between the evaluation metrics used in this study: *r* ^2^, mean bias (MD) and root mean squared deviation (RMSD) based on naïve model evaluations (upper panels) and cross‐validated model evaluations (lower panels). RMSD and MD values have been log‐transformed and extreme outliers removed. Solid points represent the evaluation metric for one of the 11 models in each dataset, based on specific‐trait models. The multiple *r* ^2^ statistic is reported for each correlation in the top right of the panel

Under naïve and cross‐validated comparisons, RMSD and MD were positively correlated, indicating that there was greater error (less accuracy) in over predictions compared to under‐predictions (Figure 5). While the three metrics were somewhat related, correlations were not consistent between naïve and cross‐validated cases (Figure 5). RMSD was correlated with both *r* ^2^ and MD in the naïve case. RMSD was correlated with MD in the cross‐validated case, but no other correlations were observed. No correlations were observed between *r* ^2^ and RMSD or MD in either naïve or cross‐validated comparisons (Figure 5). High *r* ^2^ values were not consistently associated with high accuracy or lack of bias (Figure 5).

## 4 DISCUSSION

The ability to move from descriptive to predictive science is a goal of much trait‐based research (Adler et al., 2014). Trait‐based schemes enable researchers to generalize across species. An interest in quantitatively linking traits to life history functions led to qualitative predictions of demographic rates from traits (Noble & Slatyer, 1980), and more recently, the consolidation of trait‐based studies has allowed for more quantitative predictions to be made (Falster, FitzJohn, et al., 2016). One worthy future of functional trait research is to apply trait‐based predictions to applied management problems; where resources are limited yet decisions must be made across broad suites of species. Yet, constructing and evaluating predictive models is difficult, an ecological modeler need methodological support in this endeavor. We sought to demonstrate the importance of evaluating predicting capacity when comparing non‐linear trait‐based growth models.

We showed that naïve performance metrics (i.e., those based on in‐sample model fit) ranked the “best” model differently within a given data set and in predictive tests. One could be badly misled on the performance of a model by only using naïve evaluation tools. In addition, our analysis highlighted that different metrics capture different aspects of model performance (Bellocchi, Rivington, Donatelli, & Matthews, 2010), and that the use of theory or literature to guide the selection of predictor variables increased the predictive capacity of fitted models. We expect that stronger emphasis on model predictions will identify general and transferable models, which will support, for example, predictions among ecosystems (Thomas & Vesk, 2017a).

We demonstrated the extension of growth models to generate out‐of‐sample predictions through explicit incorporation of predictor variables (Rüger et  al., 2011; Thomas & Vesk, 2017b). We used species’ traits as predictor variables because we were interested in whether species’ attributes enable predictions to new species. One might equally be interested in whether environmental conditions can predict growth (e.g., predicting growth under warmer climates) (Camac et al., 2017), in which case traits would be replaced with site attributes and cross‐validation folds would comprise distinct environmental conditions (rather than species). We found that careful selection of predictor variables, rather than the use of all available predictor variables, improved model performance under cross‐validation. Previous studies have shown that careful variable selection limits statistical “noise” in models and expressed concern for arbitrary variable selection methods that potentially neglect biological processes (Flack & Chang, 1987; Mac Nally, 2000; Warton, et  al., 2015).

As expected, naïve and cross‐validated statistics differed markedly; however, our results highlight how much worse cross‐validated metrics suggest many of the growth models are for making predictions across species based on functional traits. These differences suggest that naïve statistics do not reliably characterize a model's predictive capacity. The best‐fitting models were rarely the best predictive models. This discrepancy can be due to over‐fitting, which occurs when a model is highly flexible and fitted so well to the training data that the model is essentially fitting random noise (Olden & Jackson, 2000; Wenger & Olden, 2012). A growth curve constructed in this way might have a fabulous fit to one data set, but is not representative of new or unobserved data. When the objective of growth modeling is prediction, growth models with a certain amount of rigidity can reveal broad trends without being overly influenced by noisy data. Using a theoretically derived or ecologically relevant model can also avoid biologically implausible curves, which can occur in highly flexible models (Thomas & Vesk, 2017a). The importance of cross‐validation is likely to increase with increasing curve flexibility because flexible models present more opportunities for overfitting. As expected, the reduction in model performance going from naïve to cross‐validated cases was greater for more flexible models. For ecological systems, where data sets are often patchy, cross‐validation is a straightforward and reliable way to estimate a model's absolute performance in a predictive sense (for in‐sample or out‐of‐sample prediction).

Using multiple metrics provides detailed information on model fit, and can be a valuable tool for diagnosing problems with fitted models. For example, while RMSD and MD are closely related, they give different information on model fit. Knowing whether a model is imprecise or biased can help to determine whether that model is systematically over‐ or under‐predicting. Our growth models consistently over‐predicted, and over‐predictions were much less accurate than under‐predictions. One reason for this may be that heights are log‐normally distributed, so that there is more error at greater heights (Limpert et al., 2001). Correlation values (*r* ^2^) were not always associated with RMSD and MD, which highlights that *r* ^2^ values capture different aspects of model fit than MD (bias) and RMSD (accuracy; Figure 2). Evaluation metrics appeared to be associated less strongly in the cross‐validated case, which emphasizes the value of using multiple metrics to assess cross‐validated model performance.

It is important to align measures of model fit with a model's purpose. We would encourage testing multiple models for predictive power and choosing the model that performs the best for a given application, whether it be prediction or otherwise. Accuracy of a growth model might depend on the data type, which may depend on sample sizes or taxonomic groups (Huang et al., 1992; Zeide, 1993), so that one may not expect a single growth model to suit all data sets. While it is tempting and often encouraged to use the most common method or a model with precedent in the literature, we found that model performance differed substantially between data sets. Therefore, it is not surprising that other studies of plant‐growth models report preferences for different models, including the Chapman‐Richards (Brewer, Burns, & Cao, 1985), three‐parameter Weibull (Huang et al., 1992) and Gompertz models (Zwietering et al., 1990). Our results do suggest that sigmoidal model forms, or at least forms with upper bounds, perform better for predicting height growth of plants. In addition, three‐parameter models generally outperformed two‐parameter models.

The growmodr R package contains all models used in this study, and includes a formula interface to fit and evaluate multiple models simultaneously (see Supporting Information for example). growmodr is a collection of R functions for fitting regression models to growth curves. The emphasis is on easy model fitting and simple interfaces for extensive model comparison and model validation. All functions in growmodr are written in R 3.4.0 and use Stan 2.12.0 and rstan 2.15.1. A fitted growth model is a growmodr object and can be validated using the validate function. This function can be used to cross‐validate a fitted model or can be used to validate a fitted growth model against a holdout data set. We hope providing this code will allow others to easily test multiple growth model forms and begin validating datasets using cross‐validation.

Hooten and Hobbs (2015) called for cross‐validation, particularly out‐of‐sample predictions, to be a fundamental part of ecological analysis. The best‐fitted models do not always generalize well. In this study, we highlighted the importance and value in checking in‐sample and out‐of‐sample predictive performance and we showed that careful selection of predictor variables can reduce bias in model predictions. Hard predictive tests can be damning for ecological models because a high level of accuracy is hard to achieve. However, predictive tests do more than test predictions; they identify over‐fitted models and can give insight into whether a model captures a “true” process or is just fitting noise. We hope to encourage the use of cross‐validation or external model validation in growth model analysis by providing reproducible code and clear examples.

## CONFLICT OF INTEREST

None declared.

## AUTHOR CONTRIBUTIONS

FMT, JY, and PAV conceived ideas; FMT collected data; FMT and JY developed R code and analyzed data; FMT led writing of manuscript. All authors contributed critically to the drafts and gave final approval for publication.

## Data Availability

Open‐access datasets for all data used in this paper are available on dryad at: https://dx.doi.org/10.5061/dryad.h33db. The growmodr R package is available at https://github.com/jdyen/growmodr.
