# Supplementary material for: Using functional traits to predict species growth trajectories, and cross‐validation to evaluate these models for ecological prediction
Source: Ecol Evol. 2019 Feb 6;9(4):1554–66. doi: 10.1002/ece3.4693 (PMC6392493; doi:10.1002/ece3.4693)
Supplement: Supplementary file 1 [file ECE3-9-1554-s001.docx]

Appendix one – Thomas, Yen and Vesk

Open access datasets for all data used in this thesis are available on dryad at: http://dx.doi.org/10.5061/dryad.h33db. The growmodr R package and all source code is available at <https://github.com/jdyen/growmodr>.

# optional: clear workspace

# rm(list = ls())

# optional: set working directory

# setwd("/path/to/directory")

# install growmodr package

if (!require(growmodr)) {

  if (!require(devtools)) {

    install.packages("devtools")

  }

  devtools::install_github("jdyen/growmodr", args = "--preclean")

}

# download data from here: http://datadryad.org/resource/doi:10.5061/dryad.h33db

data_sets <- read.csv("EnsembleData_SpeciesHeights_Traits.csv",

                      row.names = 1)

# prepare data set

data.test <- data_sets[which(data_sets$Site == "MurraySunset"), ]

if (any(is.na(data.test$Ht))) {

  data.test <- data.test[-which(is.na(data.test$Ht)), ]

}

# set model settings

n.iter <- 200      # number of MCMC iterations

n.chains <- 1      # number of MCMC chains

n.cv <- "loo"      # number of cross validation folds

n.its.cv <- 200    # number of MCMC iterations for cross validation

n.chains.cv <- 1   # number of MCMC chains for cross validation

# prepare model data set

data.set <- list(size = data.test$Ht,

                 spp = as.character(data.test$Sp),

                 age = data.test$Yrs,

                 predictors = cbind(data.test$SLA,

                                    data.test$SDMASS,

                                    data.test$N))

# fit model

mod <- growmod(size ~ (age | spp / predictors),

               data = data.set,

               model = c("hillslope", "koblog"),

               n_iter = n.iter,

               n_burnin = floor(n.iter / 2),

               n_chains = n.chains,

               stan_cores = 1)

# summarise the fitted model

summary(mod)

# plot the fitted model

plot(mod)

# cross validate the fitted model

mod_val <- validate(mod, n_cv = n.cv)

# summarise the cross-validated model

summary(mod_val)
