# Supplementary material for: Using functional traits to predict species growth trajectories, and cross‐validation to evaluate these models for ecological prediction
Source: Ecol Evol. 2019 Feb 6;9(4):1554–66. doi: 10.1002/ece3.4693 (PMC6392493; doi:10.1002/ece3.4693)
Supplement: Supplementary file 2 [file ECE3-9-1554-s002.docx]

Appendix 2 – Thomas, Yen and Vesk (2017)

Table S1 – Mallee dataset raw metric values for naïve and cross-validated evaluations.


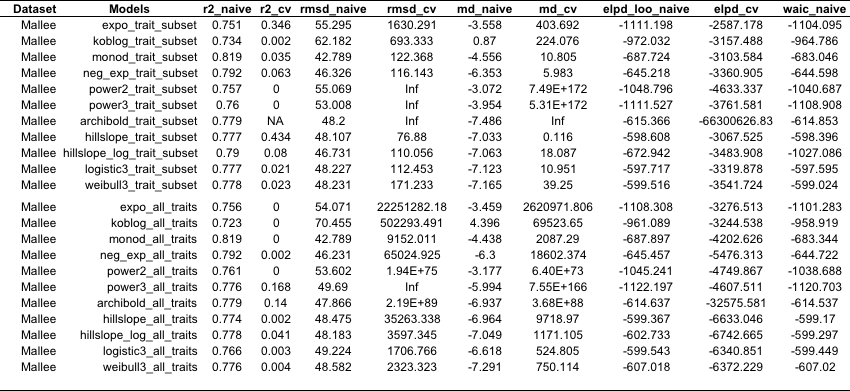


Table S2 – Myall Lakes dataset raw metric values for naïve and cross-validated evaluations.

*
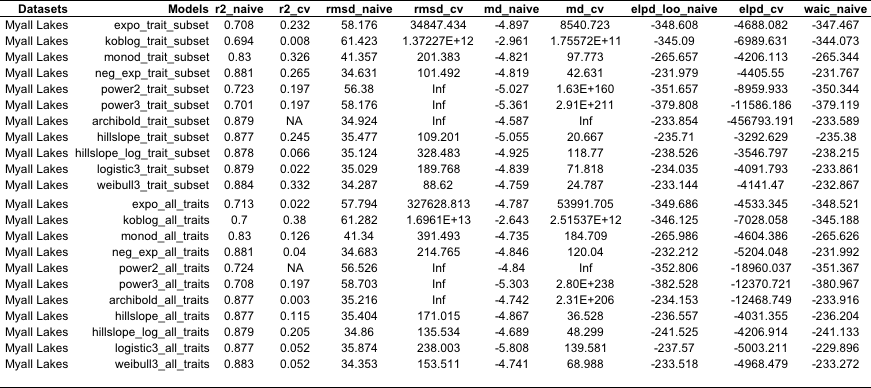
*

Table S3 – Foothills dataset raw metric values for naïve and cross-validated evaluations.

*
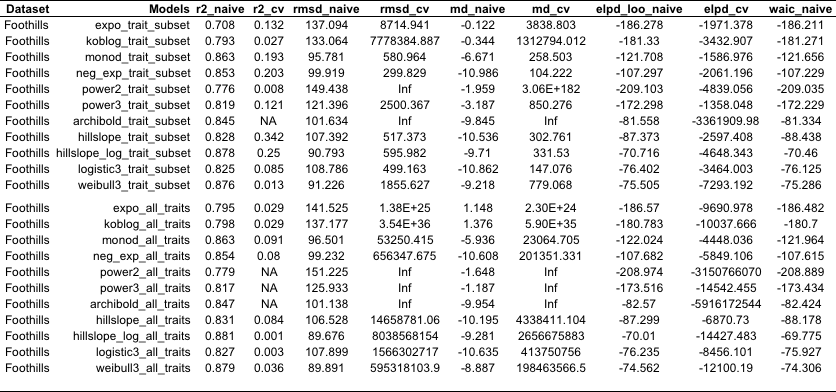
*

Table S4 – nonlinear model parameterisations. Model equations relating plant height to the three parameters are explained in the main text.

| **Model** | **Traits on *h* parameter** | **Traits on *a* parameter** | **Traits on *b* parameter** |
| --- | --- | --- | --- |
| Hillslope | SD, SLA | SD_MASS | N, SD_MASS |
| Hillslope (log) | SD, SLA | SD_MASS | N, SD_MASS |
| Power2 | SD, SLA | N, SD_MASS | *none* |
| Exponential | SLA | N | *none* |
| Monod | SLA | N | *none* |
| Negative exponential | SLA | N | *none* |
| Koblog | SLA | N | *none* |
| Power3 | SLA | SD_MASS | SD_MASS |
| Logistic3 | SLA | SD_MASS | SD_MASS |
| Archibold | SLA | SD_MASS | SD_MASS |
| Weibull3 | SLA | SD_MASS | SD_MASS |

Table S5 – modelled parameter values for each species across datasets.

| **Sites** | **Family** | **Species** | **Hmax**  **(cm)** | **a**  **(cm/cm/yr)** | **b**  **(yrs)** |
| --- | --- | --- | --- | --- | --- |
| Mallee | Mimosaceae | *Acacia bractybotrya* (16) | 146 | 3.94 | 1.82 |
| Mallee | Mimosaceae | *Acacia montana* (15) | 102 | 3.90 | 1.77 |
| Mallee | Mimosaceae | *Acacia wilhelmiana* (20) | 114 | 3.79 | 1.84 |
| Mallee | Euphorbiaceae | *Beyeria opaca* (15) | 89 | 3.30 | 2.11 |
| Mallee | Cupressaceae | *Callitris verrucosa* (15) | 322 | 3.90 | 1.56 |
| Mallee | Gyrostemnaceae | *Codonocarpus cotinifolius* (5) | 463 | 4.40 | 1.86 |
| Mallee | Sapindaceae | *Dodonaea bursariifolia* (20) | 64 | 3.95 | 1.61 |
| Mallee | Myoporaceae | *Eremophila glabra* (20) | 80 | 3.50 | 1.93 |
| Mallee | Myrtaceae | *Eucalyptus oleosa* (20) | 270 | 6.91 | 1.11 |
| Mallee | Myrtaceae | *Eucalyptus gracilis* (20) | 280 | 6.99 | 1.11 |
| Mallee | Boraginaceae | *Halgania cyanea* (20) | 42 | 4.30 | 1.31 |
| Mallee | Myrtaceae | *Melaleuca lanceolata* (15) | 222 | 4.37 | 1.68 |
| Mallee | Asteraceae | *Olearia muelleri* (16) | 62 | 3.78 | 1.70 |
| Mallee | Asteraceae | *Olearia pimeleoides* (25) | 87 | 3.95 | 1.69 |
| Mallee | Asteraceae | *Olearia subspicata* (15) | 86 | 4.10 | 1.61 |
| Mallee | Rutaceae | *Phebalium squamulosum* (15) | 58 | 3.77 | 1.69 |
| Mallee | Mimosaceae | *Senna artemisioides subsp. zygophylla* (10) | 141 | 3.97 | 1.81 |
| Mallee | Lamiaceae | *Westringia rigida* (20) | 59 | 3.41 | 1.90 |
| Myall Lakes | Mimosaceae | *Acacia suaveolans* | 173 | 1.64 | 1.51 |
| Myall Lakes | Mimosaceae | *Acacia terminalis* | 221 | 1.85 | 1.78 |
| Myall Lakes | Apiaceae | *Actinotus helianthi* | 159 | 1.44 | 2.21 |
| Myall Lakes | Fabaceae | *Aotus ericoides* | 100 | 1.07 | 1.63 |
| Myall Lakes | Proteaceae | *Banksia serrata* | 247 | 0.79 | 5.87 |
| Myall Lakes | Rutaceae | *Correa reflexa* | 81 | 1.14 | 2.54 |
| Myall Lakes | Fabaceae | *Dillwynia retorta* | 190 | 2.20 | 2.17 |
| Myall Lakes | Ericaceae | *Epacris pulchella* | 154 | 1.65 | 2.28 |
| Myall Lakes | Fabaceae | *Gompholobium latifolium* | 227 | 1.78 | 1.89 |
| Myall Lakes | Haloragaceae | *Gonocarpus teucrioides* | 69 | 1.81 | 0.81 |
| Myall Lakes | Ericaceae | *Leucopogon lanceolatus* | 51 | 0.82 | 3.72 |
| Myall Lakes | Ericaceae | *Monotoca elliptica* | 506 | 0.53 | 8.65 |
| Myall Lakes | Thymelaeaceae | *Pimelea linifolia* | 183 | 1.90 | 1.68 |
| Myall Lakes | Rubiaceae | *Pomax umbellata* | 20 | 2.54 | 0.62 |
| Myall Lakes | Euphorbiaceae | *Ricinocarpus pinifolius* | 39 | 1.14 | 1.46 |
| Myall Lakes | Elaeocarpaceae | *Tetratheca ericifolia* | 139 | 1.36 | 1.56 |
| Foothills | Mimosaceae | *Acacia oxycedrus* | 613 | 0.31 | 13.81 |
| Foothills | Mimosaceae | *Acacia verticilata* | 386 | 0.53 | 8.61 |
| Foothills | Proteaceae | *Banksia spinosa* | 378 | 0.42 | 11.14 |
| Foothills | Rubiaceae | *Coprosma quadrifida* | 470 | 0.16 | 21.27 |
| Foothills | Proteaceae | *Hakea decurrens* | 509 | 0.23 | 14.00 |
| Foothills | Asteraceae | *Olearia lirata* | 400 | 0.35 | 8.78 |
| Foothills | Rhamnaceae | *Pomaderris aspera* | 1004 | 0.43 | 12.04 |
| Foothills | Lamiaceae | *Prostanthera lasianthos* | 598 | 0.34 | 10.54 |
| Foothills | Fabaceae | *Pultanaea scabra* | 274 | 0.35 | 11.09 |
